# Supplementary material for: Further Inspection: Integrating Housing Code Enforcement and Social Services to Improve Community Health
Source: Int J Environ Res Public Health. 2021 Nov 16;18(22):12014. doi: 10.3390/ijerph182212014 (PMC8623912; doi:10.3390/ijerph182212014)
Supplement: Supplementary file 1 [file ijerph-18-12014-s001.zip › Supplemental Material.pdf]

Supplemental Material: Interview Guides

City Leadership:

I. **Overview Questions**
   a. Are there any general thoughts you'd like to share with us about your experience or perspective on the ISD-CAPIC social service referral program?
   b. In your opinion, what is the most beneficial aspect of the program?
   c. Where or what are the challenges of the program?
   d. How often do you receive updates on the referral program? How are you updated? (email, in person, monthly housing task force meetings, etc.)

II. **Program Impact**
   a. What impact have you seen, if any, on the work processes at City Hall among inspectors or other staff? On morale?
   b. Has the program impacted your work? If yes, in what ways?
   c. What impact have you perceived, if any, on residents who are in need of help?

III. **Challenges**
   a. How satisfied are you with how the program is going? (number of referrals, outcome of referrals, communication from CAPIC and inspectors, etc.)
   b. What were the challenges of transitioning to this program?
   c. What suggestions do you have to improve program?

IV. **Conditions for Success**
   a. What motivated you to try the referral program?
   b. From your perspective, what are measurements of the referral program's success?
   c. Do you think that the social service referral model between housing inspectors and community agencies could be replicated elsewhere? If yes, what kind of cities do you think would benefit most from this model?
   d. Is there anything else you would like to share?

Inspectors:

I. **Overview Questions**
   a. Are there any general thoughts you'd like to share with us about your experience or perspective on the ISD-CAPIC social service referral program?
   b. In your opinion, what is the most beneficial aspect of the program?
   c. Where or what are the challenges of the program?
   d. Can you tell me a little about the cases you referred (without mentioning names of those referred)?

II. **Impact**
   a. Can you describe the referral program's impact on how you are able to respond to families and individuals in crises that you may encounter?
   b. In your opinion, how successful has the program been in streamlining support services?
   c. How has the adoption of the program impacted your day-to-day work processes, if at all?
   d. Do you think the access to social service referrals decreased repeat inspections?
   e. How has the program impacted residents' wellbeing?
   f. Are 15 referrals about the number that you expected?

III. **Challenges**
   a. How effective is the referral program at connecting in-need residents to services?
   b. How satisfied are you with the outcomes of referred cases?

- **c.** What were the challenges of transitioning to this referral system?
- **d.** Are there any aspects of the referral program that you do not think are beneficial either to streamlining workflow, or to improving the safety/wellbeing of residents? If yes, which?
- **e.** What suggestions do you have to improve the program? Quality, relevance, efficiency?

IV. Conditions for Success

1. In your opinion, what conditions motivated ISD to adopt this type of program?
2. Do you think that the social service referral model between housing inspectors and community agencies could be replicated elsewhere? If yes, what kind of cities do you think would benefit most from this model?
3. Anything else you'd like to share?

Social Service Case Manager:

I. Overview Questions

- **a.** Are there any general thoughts you'd like to share with us about your experience as part of the CAPIC-ISD referral program?
- **b.** In your opinion, what do you personally find to be the best component of the program?
- **c.** Where or what are the challenges of the program?
- **d.** Could you talk us through how a typical referral works?
  - Who calls you? At what stage? What do you do next?
- **e.** Has the program changed the way you do your work in any way?
  - **i.** In terms of going inside people's homes? Collaborating with outside agencies?

II. Program Impact:

- **a.** Prior to the City's adoption of the referral program, how, if at all, did CAPIC interact with ISD to address social conditions that inspectors encounter while on inspections?
- **b.** In the absence of the program, do you believe residents who benefited from the social service referral program would have found CAPIC on their own? Why?
- **c.** Do you believe the collaboration with ISD allows CAPIC to provide services in a different way or to a different subset of residents? Please explain.
- **d.** How often do you collaborate with outside agencies to ensure residents get the services they need? What are the challenges of collaborating?
- **e.** What do you see as the underlying causes for referrals?

III. **Challenges**

- a. How satisfied are you with the outcomes for residents who have been referred?
  - i. Are there cases where residents were not able to get what they needed?
- b. How invested do you think CAPIC leadership and City Leadership are in the success of this program and the outcome of cases?
- c. What suggestions do you have to improve the program? Quality, relevance, efficiency?
- d. Are there any aspects of the referral program that you do not find helpful at improving the health and wellbeing of residents? If yes, which?
- e. Are 15 referrals about the number that you expected?

IV. **Conditions for Success**

- a. From your perspective, what are measurements of the referral program's success?
- b. What conditions in Chelsea motivated this program?
- c. Do you think that the social service referral model between housing inspectors and community agencies could be replicated elsewhere? If yes, what kind of cities do you think would benefit most from this model?
- d. Is there anything else you would like to share?
